# Decayed Teeth and Appendicitis; Points the Pupils of Our Public Schools Should Know Regarding Them

**Published:** 1909-03-15

**Authors:** W. Guy Smith

**Affiliations:** Cincinnati


### DECAYED TEETH AND APPENDICITIS.

### Points the Pupils of Our Public Schools Should Know Regarding Them.

BY W. GUY SMITH, D.D.S., CINCINNATI.

The problem of educating the pupils of our public schools to the necessity of oral hygiene and the care of the teeth is a hard one, to be sure. I do not stand alone in my opinion that the medium is through the school, and, while attempts have been made by progressive dental practitioners in New York, Missouri, Mississippi and Massachusetts to pass laws making it compulsory to teach these subjects in the schools, these attempts, perhaps from lack of enthusiasm, insufficient support or indifferent perseverance, or perhaps from the lack of the whole three, have proved unsuccessful.

Dr. Brown, of Chicago, says: "If the mouths of the children in our public schools could be examined by competent persons, and instruction given and enforced with regard to the intelligent use of brushes and antiseptic solutions, the death rate of this country would be materially lessened, the percentage of illness much reduced and a stronger and more vigorous race result in consequence of these prophylactic measures."

Decayed teeth are responsible for many maladies, and there is no reason why tooth-brushes should not be included in the school outfit as well as dumb-bells and other gymnastic appliances; in fact, I advocate tooth-brush drills for the children in the lower grades.

The increase of appendicitis in the last few years is indeed alarming, and it has been proven beyond a doubt that the organisms found in a septic (decayed) tooth are also found in the appendix, and there is good reason to believe that neglected teeth are largely responsible for the increase of appendicitis.

The cause of many children being dull in their studies can often be traced to decayed teeth. If the importance of correcting teeth defects at an early stage was more generally recognized by parents and teachers, we would hear much less of the harmful influences of our public schools upon the eyesight of children, and the number who pursue the course to the end would be materially increased.

Many parents, though interested in the welfare of their children in all other respects, neglect this most important duty, not appreciating the fact that a large number not up to the average in the class, or who suffer from toothache, earache, sickness, weak digestion, St. Vitus' dance, epilepsy, headache, and physical breakdown, owe their ills to some defective tooth or teeth.

A pupil should be encouraged to form regular habits. Regularity of habit, work well done, promptness, cleanliness, should be the standard, and if the child falls behind, lacks ambition, is listless, is drowsy, has symptoms of defective teeth, his parents should be urged to have him intelligently looked after and treated. Parents invariably feel grateful when interest in their children is manifested. Careful observation, intelligent care and treatment will amply repay the teacher, not only in increased learning capacity of the pupil, resulting in much less tax on the teacher's own time, patience and endurance, but he or she can enjoy the great reward of knowing he or she has helped to properly equip children for a useful life.

I believe the chief cause of nervous diseases in school children is defective teeth and abnormal oral conditions. No one can properly masticate with bad teeth, and a pupil not able to masticate properly produces abnormal conditions of the stomach, and, not developing as it should, becomes an anemic. The system being deprived of its normal functions, abnormal conditions will arise, as peculiar sensations in the head, vertigo, nausea, dyspepsia, chorea, insomnia, epilepsy, hysteria, nervous prostration, insanity, and general failure of health. Therefore, the dentist is as vital and necessary a part of family health as a family physician, and should be consulted regularly.

Seventy-five per cent. of the people wearing artificial teeth are suffering from several of the above-named ailments. One-half of the wearers of artificial teeth are regular contributors to the physician, suffering from what the patient claims is heart trouble, which is really stomach trouble caused by improper mastication and by improper insalivation; the digestive organs are called upon to do excessive work; the nerves, being the conductive power, are called upon for an extra supply of current, and by so doing takes what should go to the other parts. Hence, the paramount importance of the teeth.

In most all of our schools physiology and hygiene are taught to some extent, and I feel positive that dental hygiene will find its way into the school curriculum.

The Board of Health of New York, realizing the necessity of instruction in oral hygiene, has made arrangements for lectures to be given in the schools. Our schools have medical examiners, and why shouldn't they have dental examiners, as the mouth is the medium culture of millions of germs, some of them being most virulent.

Germany, Russia, England and Austria are making far more rapid progress than our country. The boards of health in the larger cities of the above-named countries have instituted regular inspection of the teeth of school children and in one of the principal cities in Germany a dentist gives his entire time to school work.

Every effort should be made to retain the temporary teeth until such time as nature deposes them by the eruption of the permanent set. Children should be taken to the dental office upon the first manifestation of pain, that any forming cavity may receive the attention it demands, and also that the operation may be practically painless, a feature which is especially important, as it aids in establishing that degree of confidence between the dentist and his little patient which is so much to be desired.

As soon as the incisors are in place, procure a soft-bristled tooth-brush. The teeth should be brushed from the gum toward the cutting edge to avoid irritating the gums. Rotate the brush on the grinding surface of the molars. Care should be taken to remove every particle of food from around and between the teeth after eating. Society has banished the tooth-pick from the table, but it should not thereby discourage its use, as a tooth kept perfectly clean would never decay.

One of the principal causes of decay is an acid produced usually by the decomposition of particles of food remaining in the mouth and between the teeth. The acid gradually dissolves the lime salts composing the teeth, and a cavity is the result.

Perhaps some of my readers will criticise me regarding my remarks relative to appendicitis, but after having completed my paper I discovered an article printed in a late number of the *Dental Cosmos*, by Sir Frederick Treves, which gave me implicit confidence in my assertion. Here is what he said: "Defective teeth are exceedingly common among the subjects of appendicitis, and especially among those who have passed the period of youth. Over and over again it would appear as if the want of proper and efficient teeth had been the direct cause of the attacks. Such patients often bolt their food, and such meat as they eat can hardly reach the stomach in a condition fit for complete digestion. The bolus passes into the bowels still ill-digested. It fails to stimulate normal peristalsis; is prone to lodge in the great receptacle, the cecum, where it decomposes, and if there be any existing lesion of the appendix, must tend to encourage the morbid change."

